# Strategic and entrepreneurial abilities: Surviving the crisis across countries during the Covid-19 pandemic

**DOI:** 10.1371/journal.pone.0285045

**Published:** 2023-05-03

**Authors:** Paweł Chudziński, Szymon Cyfert, Wojciech Dyduch, Salah Koubaa, Maciej Zastempowski

**Affiliations:** 1 Business Partners Club, Poznan University of Economics and Business, Poznan, Poland; 2 Institute of Management, Poznan University of Economics and Business, Poznan, Poland; 3 Department of Entrepreneurship, Faculty of Management, University of Economics in Katowice, Katowice, Poland; 4 Faculty of Law, Economics and Social Sciences, University of Hassan II, Casablanca, Morocco; 5 Department of Enterprise Management, Faculty of Economic Sciences and Management, Nicolaus Copernicus University, Torun, Poland; University of Murcia: Universidad de Murcia, SPAIN

## Abstract

This paper seeks to identify organisational abilities that influence the company’s survival during crises. To address this issue, first–through literature review–we identified five groups of crucial organisational abilities that companies pursue during the crisis, i.e., strategic, technological, collaboration, entrepreneurial and relational. We have also identified four objectives that relate to surviving the crisis. Next, we have scrutinised 226 companies from two sides of the world, Poland (Europe) and Morocco (Africa), during the Covid-19 crisis. Quantitative analysis using Structural Equations Modelling demonstrated that surviving during a crisis depends mostly on strategic and entrepreneurial abilities such as the ability to shift resources quickly, organise the work in the firm effectively and plan strategically, as well as diversify its products and services perceived as critical.

## 1. Introduction

The Covid-19 pandemic put many business organisations on the edge of survival. Managers who faced the unexpected crisis were forced to make quick, often unplanned, and unprepared decisions to secure the company operations and maintain the performance necessary for surviving the crisis that resulted from lockdowns and disrupted supply chains.

In these disturbing circumstances, questions emerge regarding the most relevant factors for a company’s survival during a crisis, as well as factors increasing the chances of continuing business operations. The Covid-19 pandemic is the longest-known crisis affecting communities and organisations worldwide; therefore, it is equally important to learn from this experience. To-date research does not fully answer the tantalising question coming from managers on how to prepare for a crisis. Our study does not provide a complete answer either. However, in our understanding, it presents yet another building block in the effort to prepare a crisis-proof enterprise. In our quest to answer the above questions, we focused on the organisational abilities that will help companies in times of crisis.

Scholars analyse the impact of past crises on the management [1], e.g. the financial crises [2], the pandemic crisis caused by the Ebola [3], and the first SARS virus [4]. There is already a strong body of evidence concerning managerial decisions during the Covid-19 crisis [5–7]. However, little is known as to which organisational characteristics are essential for companies to continue operations during crises that are difficult to predict and whose long-term impact can be significant. Specifically, this paper seeks to determine which organisational abilities influence the implementation of goals necessary for maintaining performance and surviving the coronavirus crisis. Interest in organisational abilities in two specific geographical and cultural contexts (the CEE region and North-West Africa) during Covid-19 formed the basis for this study.

The theoretical considerations are based on the following assumptions. First, we assumed that the specific abilities are aggregate variables that characterise the whole organisation, being an output of collective wisdom, organisational knowledge, and human resource management. Thus, we analyse these abilities on the organisational level and see them as strategic variables that strategic managers can use to react in unexpected situations. We do not focus on micro-foundations or individual skills or capabilities of single enterprise employees.

Second, we adopt three specific theoretical perspectives that explain the role of resources in pursuing organisational goals as a response to changes in the environment. On the one hand, we focus on the resource-based view (RBV), which assumes that every organisation is a bundle of resources, processes, routines and capabilities that—properly orchestrated–translates into firm performance. The resource-based view seeks the sources of competitive advantage in tangible and intangible assets used to implement competitive strategies [8, 9]. The RBV served here to acknowledge that, to survive the crisis, every company should be able to develop and strategically invest in a combination of valuable and scarce resources that cannot be imitated, replaced or purchased by competitors. A bundle of such resources creates an organisational skill or capability that can potentially shape firm performance. On the other hand, we look at the absorptive capacity theory that addresses resources organisations can use [10, 11]. The absorptive capacity has been defined as the capability of a given organisation to recognise the value of new external information, incorporate it and apply it to achieve business goals [11]. In other words, absorptive capacity reflects how an organisation can use knowledge existing outside of the organisation to its benefit. Thus, information seen as an intangible resource, together with other resources, can be used by organisations to realise desired strategic goals regardless of times of crises [12], which secures strategy continuity. On the third hand, when arguing about capabilities, we incorporate the dynamic capability theory (DC) [13]. The dynamic capability perspective focuses on the conscious and skilful modification of a firm’s resources and strategic potential through strategic change to achieve above-average performance [14]. The dynamic capability perspective helps to understand how bundling necessary resources during unexpected changes develops capabilities that help companies maintain their current performance. In practice, it is realised by adapting the resource base, i.e., flexible shifting of the necessary resources from current operations towards exploiting opportunities that appear or minimising threats that surprise. The ability to react correctly, adequately and timely to environmental changes requires a combination of multiple organisational capabilities. Hence, we use this perspective as we posit that multiple capabilities are needed by organisations to survive a crisis time.

Our theoretical perspective, therefore, assumes that (a) organisations require optimal orchestration of resources, processes, routines and capabilities (RBV), (b) organisations can learn and use the knowledge from outside to pursue business goals (absorptive capacity), (c) organisations can adapt to radical discontinuous changes in the environment by flexible shifting resources for exploiting opportunities or minimising threats resulting from crises.

Third, when referring to the dynamic capability theory, we assumed the delineation between substantive (operational) and strategic capabilities, focussing on the latter. The times of crisis prefer strategic capabilities that help organisations maintain their performance and sustain their operations in difficult times over a long period.

The purpose of this paper is two-fold. First, through a literature review, it attempts to identify strategically critical organisational capabilities. Second, through a survey among managers and further quantitative analysis, this paper identifies which organisational capabilities affect organisational goals essential for further survival. It should be noted that our study does not measure the actual accomplishment of goals, as it would require a longitudinal approach. We sought to identify which goals were acknowledged by managers as crucial and strategically important to survive the Covid-19 crisis, thus becoming their strategic priorities. The research was carried out from November 2020 to February 2021, during the second wave of the Covid-19 pandemic. We have collected survey data from 226 enterprises in Poland and Morocco. The obtained data were analysed using Structural Equation Modelling (SEM).

This paper contributes to the literature by demonstrating which organisational abilities affected performance during the Covid-19 crisis and identifying which goals were acknowledged by managers as most significant to survive the crisis. To our knowledge–as opposed to economic crises–not much is known about the importance of organisational abilities during pandemics, nor their influence on crucial company goals.

## 2. Theoretical underpinnings of abilities for survival

The following section identifies organisational skills and capabilities that formulate a sound capability base necessary to survive the crises. They will serve as independent variables in our framework. We argue that these capabilities influence the survival of companies during crises. Thus, the business goals oriented toward survival will constitute the dependent variables. Hence, in the following section, we discuss (1) the goals of the companies oriented for survival and (2) their abilities and competencies to implement these goals successfully.

### 2.1. Resources and abilities

The prior body of research suggests that essential elements for considering the companies’ abilities can be found in the works dedicated to competitiveness and strategy. It is emphasised that the company’s abilities are crucial in building and maintaining its competitive advantage and implementing the strategy [15, 16]. Consequently, the sources of the modern understanding of the companies’ ability to survive in a crisis time can be found, among others, in the RBV theory [8, 9], absorptive capacity theory [10, 11] or dynamic capabilities theory [13].

RBV defines an organisation as a bundle of idiosyncratic internal resources, the appropriate configuration of which can ensure that an organisation gains a competitive advantage [17] and an appropriate level of performance and survival in a crisis [18]. Xu et al. note that the resource endowment of companies determines their ability to manage, grow and cope with crises [19]. Suggesting that RBV favours large listed companies because they have better access to financial and human resources in a crisis, Grözinger et al. emphasise the importance of responding flexibly and mobilising resources [20]. Esteve-Pérez and Mañez-Castillejo suggest that a firm’s ability to develop resources enhances its ability to adapt to a changing competitive environment and improves its survival prospects [21]. Chatzoudes et al.’s findings show the indirect impact of RBV factors on a company’s long-term survival during an economic crisis [22]. Mayr et al. show the desirability of applying RBV assumptions in the context of crisis-affected companies [23]. Salunkhe et al. suggest that using RBV in critical business transformations can ensure a company’s survival during a pandemic by supporting customer, employee and resource engagement [18]. In our research, following Bhattacharyya and Thakre, we recognise the usefulness of an integrated RBV and dynamic capabilities approach in a socio-economic crisis, assuming that while RBV enables initiatives to build resources to counter the impact of the COVID-19 pandemic, DC-based thinking allows us to determine what resources and capabilities will be needed during a crisis [24]. Dynamic capabilities, which emphasise the need to integrate and reconfigure internal resources in a changing business environment [25], are considered an extension of RBV theory [26]. Jiang et al. point out that how an organisation responds to and recovers from a crisis/disaster depends on its DCs, which enable it to learn and adapt to a turbulent environment [27]. Mansour et al. research indicates the importance of shaping crisis management capabilities, including dynamic and adaptive capabilities [28]. Findings from Duarte et al. show that companies that survive a crisis have developed new adaptive capabilities, including the ability to shorten existing power distance, replace scarce resources, retain key talent and obtain alternative sources of funding [29]. Jibril et al., pointing to the role of shaping absorptive capacity in a crisis, emphasise the importance of activities related to organisational learning processes that allow for building organisational resilience [30].

Embedded within RBV, DC and absorptive capacity theories, scholars offer different typologies of resources, skills and competencies: six critical categories of resources: financial, physical, human, technological, reputational and organisational [31]; others mention five main types of substantive capabilities and six categories of higher- order dynamic capabilities [32]. On the other hand, when examining innovative abilities, the following groups of capabilities are indicated: learning, R&D, resources allocation, manufacturing, marketing, organising, and strategic planning [33].

Referring to the scholarly discussion [13, 34–37], some categories of resources and capabilities can be identified [38]: technological, financial, human, reputational, structural, institutional, and market resources and skills. Based on various categorisations from the literature review, we have identified 14 abilities that organisations use during crises: diversification of goods and services, ability to shift resources quickly, effective planning and work organisation, using technological advancements, building efficient IT systems, ability to work in virtual teams, delegating responsibilities for empowerment and autonomy of employees, using personal contacts, ability to benefit from appearing opportunities, ability to innovate, ability to start new business/venture, ability to access sources of financing, ability to change suppliers quickly and ability to find new customers quickly. We decided to bundle different abilities and classify them into the following groups of abilities that can be seen as the important ones in the context of the Covid-19 crisis: (1) strategic, (2) technological, (3) collaboration-oriented, (4) entrepreneurial (including innovation-market connection) and (5) relational (see Table 1). Below, some theoretical underpinnings concerning each group are given.

**Table 1:** Survival abilities.

Strategic abilities	Diversification of products and services offered so far
	Ability to shift resources quickly
	Effective planning and work organization
Technological abilities	Using technological advancements
	Building efficient IT systems
Collaboration abilities	Ability to work in virtual teams
	Delegating responsibilities for empowerment and autonomy of employees
	Using personal contacts
Entrepreneurial abilities	Ability to benefit from the opportunities appearing in the crisis
	Ability to innovate
	Ability to start new businesses
Relational abilities	Ability to access sources of financing
	Ability to change suppliers quickly
	Ability to find new customers quickly

#### 2.1.1. Strategic abilities

During the Covid-19 pandemic, businesses faced sudden and unexpected governmental decisions that forced them to respond adequately and introduce measures that wholly or slightly changed the business model and activities. Organisations had to run their businesses differently, and processes had to change rapidly for organisations to survive. In response to the crisis, new products or services had to be offered, which required quickly shifting resources to exploit the opportunity arising or minimise the threats [39] and create value [34]. During crises, entrepreneurial opportunity pursuit requires focusing managerial attention and necessary organisational resources from already exploited opportunities towards newly-recognized opportunities [40]. Resource orchestration seeks to accumulate, combine, and use resources to support current opportunities and create future opportunities through diversification, specialisation or innovation [41].

By sense-making and sense-giving, any event appearing in uncertainty can be identified as an opportunity [42], and agile firms adapt quickly to exploit opportunities [43]. However, scholars posit that traditional models of responding and adapting to changing environments may not be fully adequate during unexpected crises [44]. Therefore, identifying the opportunity under a limited period and shifting resources to these places of an organisation requires an agile approach with key employees involved, as well as benefiting from the bricolage and effectuation [45].

In turbulent environments, more organisations can develop resilience and survive through a proper human resource management [46], creating the workforce agility [47] or introducing flexible forms of work [48].

It is also posited that effective work organisation matters in developing a resilience [49], but it requires good managerial and strategic moves such as effective resource, work and organisation planning [50–54], as well as developing strategic innovativeness based on the ability to shift resources quickly [15, 55–57].

The above considerations lead us to formulate the following hypothesis:

*H*_*1*_*—Strategic abilities positively impact the company’s survival during the Covid-19 crisis*.

#### 2.1.2. Technological abilities

The demand for technological capacity building determines technology development in organisations—positively driven by a firm’s market growth prospects–as well as by higher levels of competition in both domestic and foreign markets by the rate of technology. Exploring advanced technologies reduces the cost of future product innovation, allowing companies to increase market response speed, changing customer preferences and competitive changes [58].

The ability to develop new technologies helps to increase or at least maintain market share in several fields, e.g. robotic technology in the health service [59], LCD production [60] or shrimps production [61]. Using technological advancements is an ability that can become a significant part of the company’s strategy as it facilitates dealing with greater customer expectations, assuring timely and better service, and mitigating increased competitive pressures [62]. Using technology advancements to create value for customers and investing in technology development are positively correlated with growing market share [63, 64], and with firm performance [65], specifically when firms offer their products on international markets [66].

Consequently, we formulate the following hypothesis:

*H*_*2*_*—Technological abilities positively impact the company’s survival during the Covid-19 crisis*.

#### 2.1.3. Collaboration-oriented abilities

The collaboration-oriented abilities embrace the ability to work in virtual teams, delegate responsibilities for the empowerment and autonomy of employees, and use personal contacts (social capital) to increase resilience and increase the chance of company survival.

Although research on the role of cooperation in ensuring organisational resilience focuses mainly on issues related to supply chains, some scholars refer to internal collaboration and the ability to cooperate with key stakeholders as it helps reach common outcomes [67], enhancing above-average performance [68], adapting to dynamic environments [69], and improving responsiveness while mitigating effects of disruption [70]. Collaboration-oriented abilities may be seen as first-order dynamic capabilities, including team building, project management, and interpersonal communication skills [71].

The Covid-19 pandemic forced an increase in the virtual work [72] and the fast introduction of flexible working models [73], where developed relationships, shared understanding, and trust serve as essential antecedents of virtual collaboration and success of virtual teams [74], given that technical skills, and efficient IT systems enabling to switch to virtual teamwork are assured [75].

In emergencies, delegating tasks increases perceived productivity and higher manager quality [76, 77]. However, some scholars argue that organisational resilience leads to more power delegation and employee participation in the decision-making [78].

Finally, personal contacts, as a form of social capital, can help organisations resist or even avoid crises [79–82].

Hence, we formulate the following hypothesis:

*H*_*3*_ *–Collaboration-oriented abilities positively impact the company’s survival during the Covid-19 crisis*.

#### 2.1.4. Entrepreneurial abilities

Entrepreneurship plays a crucial role during unexpected external shocks, and entrepreneurial firms are more prone to survive crises [83]. During crises, as a vital portion of income is lost, managers look for opportunities that include rejuvenating or even reinventing their firm’s [84] and seek to invest time in building both potential and realised absorptive capacity, as these define greater innovation success [85] and thus greater resilience to crisis. Entrepreneurial abilities are broadly defined as abilities to start or run a business involving innovation, risk-taking added value [86].

The companies that implement bricolage entrepreneurial responses can ease the crisis effects [87] or difficulties concerning the availability of financing new businesses [88]. The strategies adopted by small firms during crises include flexible human resource practices, cost reduction, enhancing customer relations, using government support schemes, and engaging more with internal stakeholders [89], as well as learning for resilient performance [90] or improving finance, strategy and adapting to the institutional environment [91].

The past crises seem to have changed the relationship between innovation and firm performance [92]: the role of firm resources after the recession has strengthened, while the uncertainty and barriers to innovations increased. Recent research indicates that firms under the Covid-19 pandemic are innovating their marketing strategies, modifying their resource dependency and developing dynamic capabilities to respond to environmental changes effectively [93].

Every crisis affects entrepreneurial activity since it forces entrepreneurs to act under high uncertainty, resource scarcity and a high probability of failure. Entrepreneurs, therefore, are exposed to economic volatility on the one hand, as well as sensing and exploiting opportunities on the other hand [94]. Entrepreneurial activity is a primary driver of economic recovery and growth in the post-recession time [95]. Even in the tourism sector, scholars see the Covid-19 crisis as an opportunity to redirect the branch and move forward by adopting a more sustainable path [96]. The consequences of the Covid-19 crisis will likely trigger changes in work digitalisation, thus reducing mobility needs and energy consumption [97]. The Covid-19 crisis can be a source of opportunities for development in other branches, e.g. healthcare [98], waste management [99], and education [100]. It can become an opportunity window for developing sustainability transitions in various branches, but it can be achieved with careful strategic planning [101]. In the wake of Covid-19, entrepreneurs, intentionally or not, are engaging in new activities and, new behaviours, new ways of doing things, which can potentially drive business growth and contact with others that face-to-face and during a pandemic remotely bring new opportunities to explore and possibly exploit [102].

Consequently, we formulate the following hypothesis:

*H*_*4*_*—Entrepreneurial abilities positively impact the company’s survival during the Covid-19 crisis*.

#### 2.1.5. Relational abilities

We posit that relational abilities during crisis times comprise the ability to access sources of financing, the ability to change suppliers quickly, and the ability to find new customers quickly.

Access to sources of financing became crucial for companies during unexpected crises [103]. Supply of bank finance remains critical to firm investment during the crisis [104]. In the aftermath of the financial crisis, access to external finance for SMEs is a critical issue [105].

Unfortunately, during the crises, access to external sources of financing is much easier for larger companies, groups, or majority foreign-owned firms [106]. External finance access depends on internal and external factors, including legislation [107]. Internal and external factors, company growth, ownership, age and size of the firms are important variables influencing the ability to access financing before and after the crisis [105]. Managerial talent seems essential in gaining access to financial resources that increase firm value [108, 109].

Crises (e.g., caused by dangerous atmospheric phenomena or pandemics) may cause supply chain collapse resulting in serious production and financial difficulties. The role of the resources and competencies, including dynamic ones, is to adequately respond to crises, mainly by looking for new business opportunities (new suppliers, recipients), reconfiguring the resource base, and taking advantage of opportunities during a crisis [110].

In light of the above considerations, it is possible to formulate the following hypothesis:

*H*_*5*_*—Relational abilities positively impact the company’s survival during the Covid-19 crisis*.

### 2.2. The survival-oriented goals

During crises, companies set various goals to survive, which become immediate challenges during crises. Previous research [4, 111–120] indicates that the most frequent goals oriented for survival and resilience include the following: (1) keeping the current personnel, (2) maintaining positive cash flow, (3) maintaining revenues at the current level, and (4) maintaining market share. As we have developed these dependent variables in the previous research [14], we only briefly mention them for space and parsimony reasons.

#### 2.2.1. Keeping the current personnel

During crises, shortages of human resources for implementing an organisation’s tasks are particularly noticeable [121]. The lack of critical human resources was visible during the Covid-19 pandemic and resulted in breaking the supply chain, shortages in delivery or job absenteeism [122]. Keeping the current personnel is a challenge for human resources management and requires dynamic reactions, as well as prior strategic actions [123]. Retaining employees, particularly skilled employees, is a strategic condition for survival during crises [124], as well as after it.

#### 2.2.2. Maintaining a positive cash flow

Contemporary companies are able to sense changes in the market [125] and exploit opportunities by bundling and reconfiguring the necessary resources and competencies. However, exploiting opportunities, innovating, and product development do not necessarily translate into positive cash flow, as these are long-term commitments. Organisational capabilities translate into maintaining money flow and efficiency in the long run [126], but it is not simple to identify which abilities influence sustaining the cash flow [13]. In the long run, we posit that maintaining positive cash flow is one of the organisations’ critical goals.

#### 2.2.3. Maintaining revenues at the current level

One of the significant strategic goals the companies need to pursue is keeping the revenues mostly intact. The literature demonstrates that the optimal selection of organisational abilities can influence revenues [127]. However, this influence depends on the dynamism and heterogeneity of the firm environment [128]. As the environmental dynamism varies significantly during crises, it is critical for organisations to maintain revenues at some sort of stable level during unexpected disturbances by flexible resource allocation, diversification, adaptation to changes, and finding new channels in supply chains (new distributors, new buyers, etc.).

#### 2.2.4. Maintaining market share

Unexpected crises have a negative impact on the level of production and, as a consequence, on sustaining the market share, as supply chains are typically damaged by crises [129], which was evident during the Covid-19 crisis [14, 130]. What was more, organisations needed to care for human resources to maintain the production capacity unchanged and, as a result, maintain their market share [111]. Hence, while expanding the market share can be seen as a strategic goal for regular times, maintaining the market share can be seen as a strategic goal for crisis times.

### 2.3. The conceptual model

**Fig 1:**
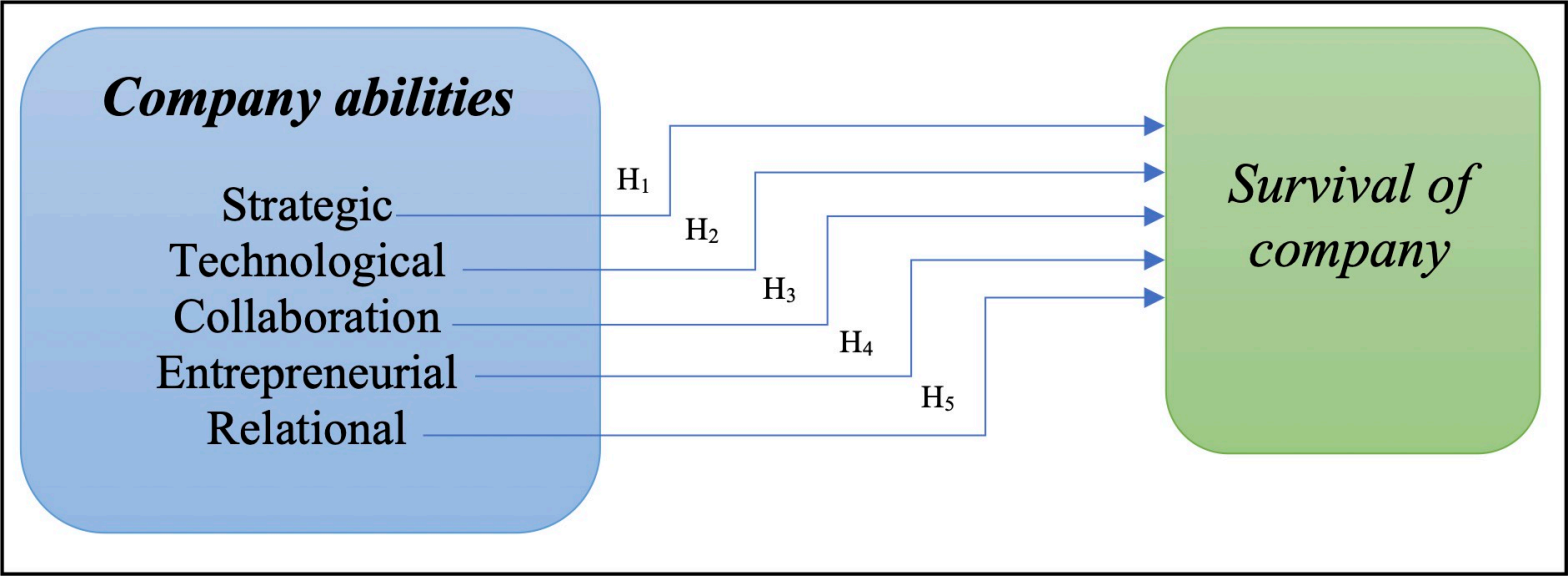
Conceptual model.

The above analysis of research conducted so far in the field of possible abilities that may impact the company’s survival during the Covid-19 crisis enabled us to formulate the following conceptual model (Fig 1).

## 3. Empirical analysis

### 3.1. Data collection and sample

The data presented in this study come from empirical research carried out from November 2020 to February 2021, during the second wave of the Covid-19 pandemic. Its purpose was to identify which kind of enterprise abilities are essential from the perspective of surviving during crisis time. We have collected our data from enterprises in two countries: Poland and Morocco (Table 2). The principal part of the research was conducted using the CAWI method. The final dataset is made up of 226 observations. All respondents gave their informed consent (oral) to participate in the study.

**Table 2:** Morocco and Poland–comparison of economic data– 2020.

Country	GDP—current USD (millions)	GDP growth (annual % 2020/2019)	Unemployment, total (% of total labour force)	Employment to population ratio, 15+, total (%)	Inflation, GDP deflator (annual %)
Morocco	114725.07	-6.3	10.2	38	0.9
Poland	596624.36	-2.5	3.5	55	4.1

Source: The Word Bank. Data.

The characteristics of the surveyed companies are presented in Table 3. As can be seen, the vast majority conducted activities in Poland (65.5%), belonged to the medium-sized enterprise group (35.4%) and had been operating on the market for between 21 and 30 years (38.5%).

**Table 3:** The structure of the sample.

Characteristics	Sample (%)
*Country*	
Morocco	34.5
Poland	65.5
*Firm size (no*. *of employees)*	
< 9	9.7
10–49	25.7
50–249	35.4
250 and more	29.2
*Age of firm (years)*	
< 10	16.8
11–20	17.7
21–30	38.5
> 31	27.0

### 3.2. Variables

Table 4 presents the description and labels of all variables included in the modelling process. We grouped them around the proposed constructs: strategic ability, technological ability, collaboration ability, entrepreneurial ability, relational ability and survival of the company.

**Table 4:** Summary of variables.

Constructs	Description	Label
Strategic abilities	Diversification of products and services offered so far	Ability 1
	Ability to shift resources quickly	Ability 2
	Effective work organization and proper planning	Ability 3
Technological abilities	High level of technological advancement	Ability 4
	Efficient IT systems	Ability 5
Collaboration abilities	Ability to work in virtual teams	Ability 6
	Delegating responsibilities and greater autonomy of employees	Ability 7
	Personal (individual) contacts	Ability 8
Entrepreneurial abilities	Ability to benefit from the opportunities appearing in the crisis	Ability 9
	Ability to innovate	Ability 10
	Ability to start new businesses	Ability 11
Relational abilities	Easy access to sources of financing	Ability 12
	Ability to change suppliers quickly	Ability 13
	Ability to find new customers quickly	Ability 14
Survival of company	Keeping our current personnel	Survive 1
	Maintaining positive cash flow	Survive 2
	Maintaining revenues at the current level	Survive 3
	Maintaining market share	Survive 4

The survey questions were measured through the seven-point Likert scale used to indicate the degree of importance of the variables. The scale ranged from 1 (‘entirely disagree’) to 7 (‘entirely agree’). Regarding variables related to the company’s abilities, respondents were asked to assess the importance of the specific abilities for surviving the crisis. In turn, in the case of variables related to the company’s survival–the importance of the specific goals to the company’s survival. The descriptive statistics of all variables are presented in Table 5.

**Table 5:** Description of variables (n = 226).

Variables	Mean	S.E.	S.D.	SD^2^
Ability 1	3.81	0.150	2.260	5.109
Ability 2	4.36	0.139	2.091	4.373
Ability 3	5.74	0.106	1.588	2.523
Ability 4	4.61	0.138	2.076	4.310
Ability 5	5.71	0.117	1.754	3.077
Ability 6	4.41	0.142	2.128	4.527
Ability 7	4.16	0.130	1.947	3.791
Ability 8	4.67	0.133	2.002	4.008
Ability 9	4.42	0.144	2.161	4.671
Ability 10	4.62	0.142	2.132	4.547
Ability 11	4.31	0.147	2.211	4.890
Ability 12	5.04	0.134	2.011	4.042
Ability 13	3.86	0.137	2.053	4.217
Ability 14	4.07	0.147	2.213	4.898
Survive 1	5.89	0.100	1.511	2.282
Survive 2	6.27	0.080	1.206	1.453
Survive 3	6.13	0.088	1.330	1.769
Survive 4	5.52	0.128	1.928	3.717

### 3.3. Methods

The obtained data were analysed using structural equation modelling (SEM) since there is little knowledge of how the variables are related to the company’s survival during the Covid-19 crisis. The aim of our research was to predict and explain survival through entrepreneurial and strategic abilities. We have collected our data from 226 enterprises. The research model used to analyse these data deals with 18 items (14 for abilities and 4 for survival). Partial Least Squares Path Modelling (PLS-SEM) seems well adapted to our research objective based on these two descriptive characteristics, sample size and several items. Moreover, PLS-SEM is used to relax some strong assumptions of Covariance-Based SEM (CB-SEM) [131] related to sample size and distribution when researchers analyse complex models with simultaneous relationships [132]. By using PLS-SEM, there are no identification issues with a small sample size (226 enterprises) which can achieve high levels of statistical power. The precision can be improved with a larger sample size. This technique is a non-parametric method with no distributional assumptions. In addition to that, our data are not normally distributed. Then, it’s impossible to use the CB-SEM approach [131]. This research is neither confirmatory nor exploratory. It focuses on the interplay between prediction and theory testing [133].

The systematic assessment of PLS-SEM results follows a two-step process: (1) known as measurement model; the first process involves an evaluation of the relationship between constructs and their items; (2) the second process, known as a structural model, involves the significance of the relationships between constructs.

To analyse the data, we used the SmartPLS software. The results of our research will be presented as follows: the measurement model and the structural model.

## 4. Results

### 4.1. Assessing the measurement model

The measurement model shows the relationship between each latent variable and its items. All the items in our questionnaire are reflective, not formative. Before analysing the relationships between latent variables, checking if the measurement model meets all the required criteria is essential. Reliability, convergent validity and discriminant validity are the most important for assessing the measurement model. The results are presented in Table 6.

**Table 6:** Statistics of the measurement model.

Constructs	Cronbach’s Alpha	rho_A	Composite Reliability (CR)	Average Variance Extracted (AVE)
Entrepreneurial abilities	0.904	0.918	0.940	0.838
Relational abilities	0.629	0.681	0.792	0.562
Collaboration abilities	0.750	0.755	0.860	0.673
Strategic abilities	0.736	0.817	0.841	0.639
Surviving	0.731	0.756	0.820	0.536
Technological abilities	0.758	0.759	0.892	0.805

As shown in Table 6, all the composite reliability values (CR) are above 0.70. Thus, the reliability is satisfactory. However, the CR of entrepreneurial abilities is above 0.90 and close to 0.95. So, it is not desirable because it indicates that all the items measure the same phenomena and are not likely to be valid measures of the construct. Even with this undesirable value, the measurement is kept in the model because it will be used as a second-order construct.

The values vary from 0.792 for relational ability’s construct to 0.94 for entrepreneurial ability. Consequently, these required criteria are satisfied. Cronbach’s Alpha values confirm the internal consistency of all scales used to measure the constructs, except the low value of the relational abilities construct [131].

A common rule of thumb is that the loadings should be 0.7 or higher [132, 134, 135]. The PLS output results show that most loadings are above 0.707. Few of them equal 0.7 (Ability 14 = 0,699: *Ability to find new customers quickly*; Survive 4 = 0.670: *Maintaining market share*; Survive 3 = 0.688: *Maintaining revenues at the current level*). Another established rule of thumb is that a latent variable should explain a substantial part of each indicator’s variance, usually at least 50%. This means the variance shared between the construct and its indicator is larger than the measurement error variance. The AVE’s indicator, Average Variance Extracted, is used to evaluate the convergent validity.

As indicated in Table 6, all the values provided by PLS-SEM output are larger than 0.50. They suggest that, on average, each construct explains more than half of the variance of its items. Two measures were used to assess the discriminant validity: the cross-loading matrix (Table 7) and the Fornell-Larcker Criterion (Table 8).

**Table 7:** Matrix of factor loadings (cross-loadings, n = 226).

	Entrepreneurial abilities	Relational abilities	Collaboration abilities	Strategic abilities	Surviving	Technological abilities
Ability 1	0.719	0.480	0.567	0.714	0.217	0.571
Ability 2	0.683	0.516	0.599	0.821	0.244	0.711
Ability 3	0.510	0.415	0.517	0.857	0.407	0.662
Ability 4	0.743	0.553	0.663	0.728	0.276	0.903
Ability 5	0.569	0.508	0.685	0.716	0.262	0.891
Ability 6	0.632	0.569	0.873	0.523	0.212	0.663
Ability 7	0.679	0.608	0.873	0.588	0.234	0.665
Ability 8	0.492	0.380	0.704	0.556	0.214	0.510
Ability 9	0.914	0.637	0.639	0.614	0.276	0.591
Ability10	0.933	0.630	0.719	0.749	0.308	0.763
Ability11	0.899	0.578	0.664	0.704	0.238	0.651
Ability 12	0.405	0.833	0.422	0.389	0.353	0.407
Ability 13	0.511	0.709	0.557	0.452	0.208	0.511
Ability 14	0.702	0.699	0.526	0.485	0.208	0.468
Survive 1	0.101	0.105	0.110	0.229	0.670	0.146
Survive 2	0.213	0.326	0.192	0.340	0.855	0.256
Survive 3	0.014	0.156	0.095	0.071	0.688	0.086
Survive 4	0.389	0.342	0.303	0.348	0.700	0.284

**Table 8:** Matrix of correlations between latent variables, Fornell-Larcker Criterion (n = 226).

	1	2	3	4	5	6
1. Entrepreneurial abilities	**0.916**					
2. Relational abilities	0.673	**0.749**				
3. Collaboration abilities	0.737	0.638	**0.821**			
4. Strategic abilities	0.753	0.566	0.681	**0.800**		
5. Surviving	0.302	0.360	0.269	0.387	**0.732**	
6. Technological abilities	0.734	0.592	0.751	0.805	0.300	**0.897**

The former indicates that an indicator’s outer loadings on the associated construct should be higher than any of its cross-loadings on the other constructs. This rule of thumb is required in our model, as shown in Table 7. The items, Ability 1, Ability 2, and Ability 3 load high on their construct strategic abilities but much lower on the other constructs. The same result can be viewed for each group of items associated with their construct. The latter is given in Table 8, the matrix of correlations between latent variables and the square root of the AVE values. The square root of each construct’s AVE should be larger than its highest correlation with any other construct. The square root of the AVE gives the values in the diagonal matrix.

PLS results show that all constructs’ AVE values are higher than 0.50. This means that each latent variable explains at least 50% of the variance of its items. Table 6 shows that for all latent variables, AVE > 0.5 and Table 7 shows that almost all indicator’s outer loading exceeds 0.7. These two essential conditions lead to the conclusion that convergent validity is established.

### 4.2. Assessing the structural model

Once the measurement model assessment is satisfactory, structural relationships between constructs should be tested. The structural model provides the empirical answer to the research question. In this article, we want to show which abilities impacted firms’ survival during the Covid-19 crisis. Based on the factor analysis, we can identify five abilities: technological, entrepreneurial, strategic, collaboration, and relational abilities.

Three standard assessment criteria are usually used to evaluate the structural model: the coefficient of determination R^2^, the blindfolding-based cross-validated redundancy Q^2^, and the statistical significance and relevance of path coefficients [131]. However, collinearity must be examined using the VIF values, which should be close to 3 and lower [131] or even below 5 [132].

All VIF values are close to 3 and below the threshold of 5 (Table 9). Therefore, collinearity among the predictor constructs is not a critical issue in the structural model; we can continue our assessment of the structural model by examining the results report.

**Table 9:** Inner VIF values.

	*1*	*2*	*3*	*4*	*5*	*6*
*1. Entrepreneurial abilities*					3.343	
*2. Relational abilities*					2.000	
*3. Collaboration abilities*					2.916	
*4. Strategic abilities*					3.394	
*5. Surviving*						
*6. Technological abilities*					3.718	

**Fig 2:**
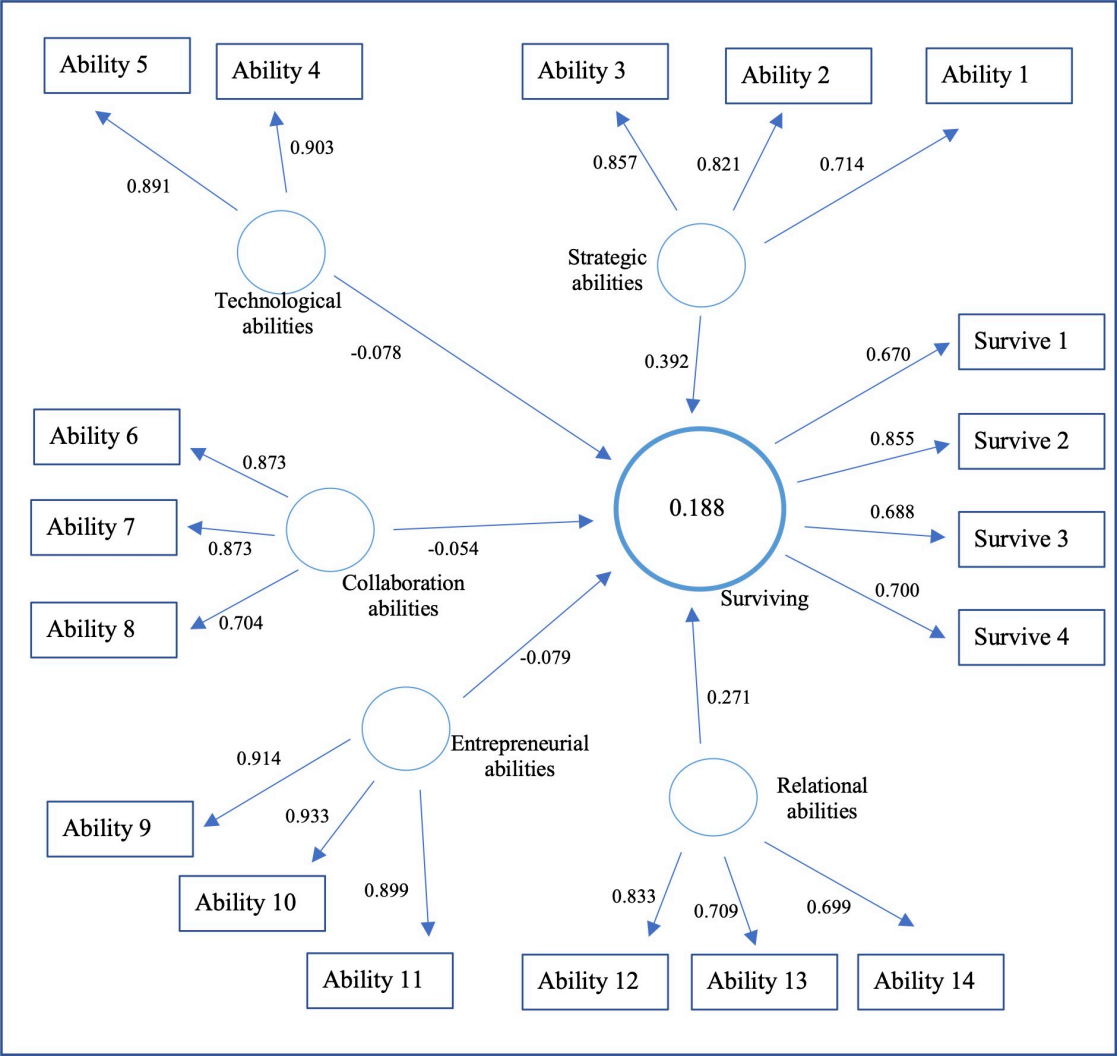
Path modelling results.

To begin with, we look into the R^2^ value of our endogenous latent variable, surviving. Based on our rule of thumb, the R^2^ value of surviving (18,8%) can be considered weak. However, as stated by Moksony, the small value of R^2^ might be beautiful in social research, especially when we look for the impact of only a few independent variables rather than a complete list of the dependent variables [136]. Later, we will try to improve the explanatory power of our model by introducing Strategic Entrepreneurial Abilities as a second-order analysis. With the path modelling results (Fig 2), we can see the explained variable’s path coefficients and R^2^ values.

To obtain the effect size *f*^*2*^ for all structural model relationships, the PLS results report provides the effects for combinations of surviving and corresponding exogenous constructs. There is no effect for surviving and its corresponding predictor’s entrepreneurial abilities, collaboration abilities, and technological abilities, whereas the effects of surviving on strategic and relational abilities are considered weak (Table 10).

**Table 10:** The effect size *f*^*2*^.

	*1*	*2*	*3*	*4*	*5*	*6*
*1. Entrepreneurial abilities*					0.002	
*2. Relational abilities*					**0.045**	
*3. Collaboration abilities*					0.001	
*4. Strategic abilities*					**0.056**	
*5. Surviving*						
*6. Technological abilities*					0.002	

PLS results report also provides an overview of the total effects given by the path modelling results. As shown in Fig 2, strategic abilities have the strongest total effect on survival (0,392), followed by relational abilities (0,271). Collaboration (-0,054), technological (-0,078), and entrepreneurial (-0,079) abilities do not have an important total effect on surviving. Diversification of products and services, proper work organisation and planning, and the ability to quickly shift resources are important to survive a crisis. Moreover, abilities such as access to financial resources, quickly changing suppliers, and finding new customers have an important effect on the company’s survival.

We run the bootstrap procedure to check if these structural relationships are statistically significant. Assuming a 5% significance level, we find, in Table 11, that two relationships in the structural model are significant (Strategic abilities → surviving (T-value: 2,814), Relational abilities → surviving (T-value: 2,552)). This supports H_1_ and H_2_. Other structural relationships have lower values, less than 1,96 (Collaboration abilities → surviving (T-value: 0,523), Entrepreneurial abilities → surviving (T-value: 0,570), and Technological abilities → surviving (T-value: 0,634)). Therefore, H_2_, H_3_ and H_4_ are rejected. These results state that strategic and relational abilities are the most important for survival during a crisis. This is not surprising, considering the importance of diversification and the ability to change and find quickly other resources or even new suppliers and customers.

**Table 11:** Path coefficients, mean, STDEV, T-values, P-values.

	*Original Sample (O)*	*Sample Mean (M)*	*Standard Deviation*	*T Statistics*	*P Values*	*Significance (p<0.05) or T>1,96*
*Entrepreneurial abilities -> surviving*	-0.079	-0.089	0.138	0.570	0.569	No
*Relational abilities -> surviving*	0.271	0.280	0.106	**2.552**	**0.011**	**Yes**
*Collaboration abilities -> surviving*	-0.054	-0.030	0.102	0.523	0.601	No
*Strategic abilities -> surviving*	0.392	0.388	0.139	**2.814**	**0.005**	**Yes**
*Technological abilities -> surviving*	-0.078	-0.077	0.124	0.634	0.526	No

Finally, to assess the predictive relevance of our model, we should run the blindfolding procedure under PLS-SEM software. The results report gives us the construct cross-validated redundancy estimates. Table 12 presents the summary of the total outcomes of the blindfolding procedure. It shows SSO as the Sum of the Squared Observations, SSE as the Sum of the Squared prediction Errors, and 1-SSE/SSO as the value of *Q*^*2*,^ which is used to judge the model’s predictive relevance about surviving as an endogenous variable.

**Table 12:** The model’s predictive relevance.

	*SSO*	*SSE*	*Q^2^ (= 1-SSE/SSO)*
*Entrepreneurial abilities*	678.000	678.000	
*Relational abilities*	678.000	678.000	
*Collaboration abilities*	678.000	678.000	
*Strategic abilities*	678.000	678.000	
*Surviving*	904.000	842.785	0.068
*Technological abilities*	452.000	452.000	

As shown in Table 12, the *Q*^*2*^ value of surviving is positive and close to zero (0,068). Then the predictive relevance of the model is not so high. That’s why it could be relevant to introduce the second-order analysis to improve the explanatory power, *R*^*2*^, and the predictive relevance of our model, *Q*^*2*^.

### 4.3. Improving the explanatory power by second-order analysis

**Fig 3:**
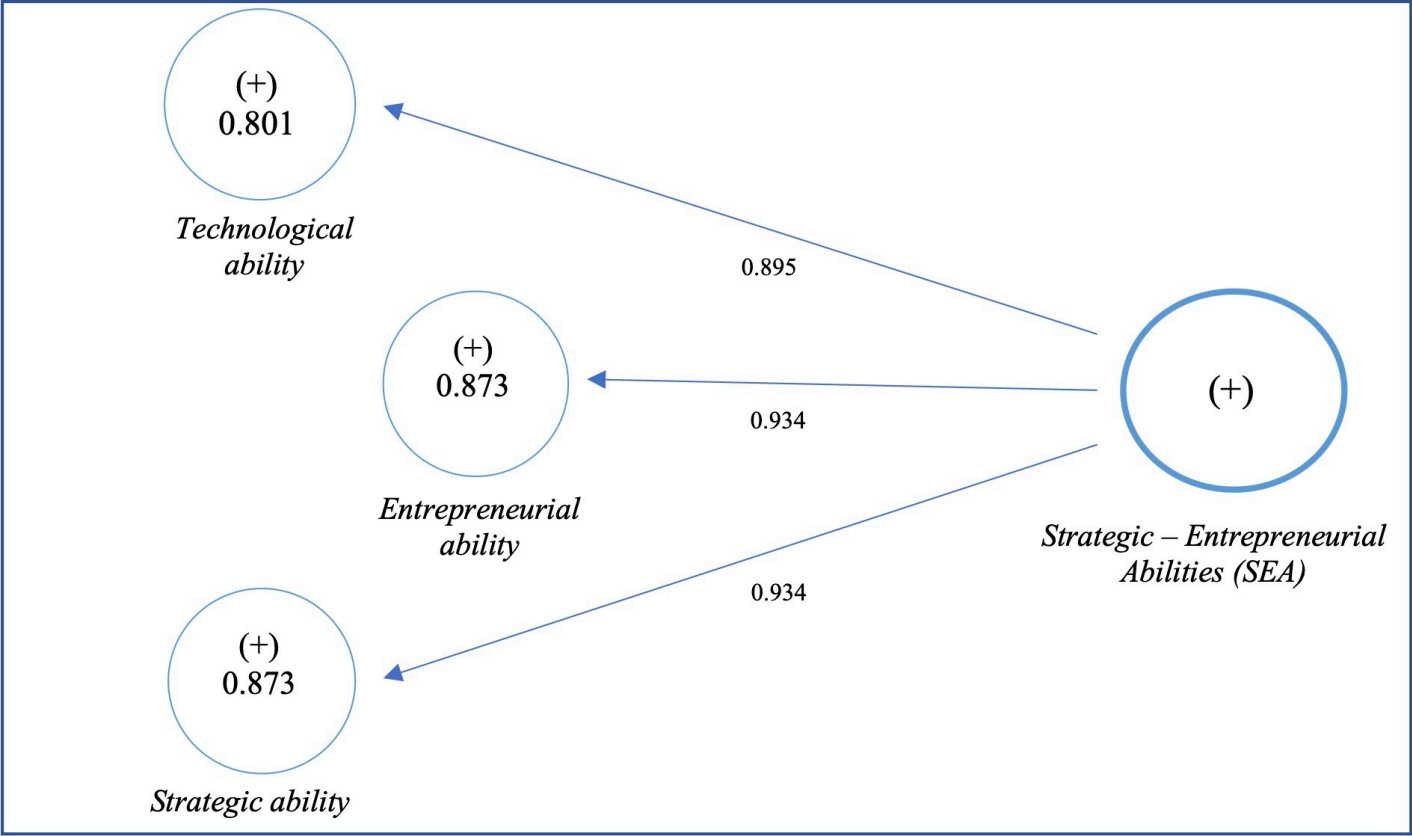
SEA as a second-order construct.

Based on the correlation matrix (Table 8), a second-order model, also called a hierarchical or second-order model, has to be created [137]. We labelled it: Strategic-Entrepreneurial Abilities (SEA) (Fig 3), which is a second-order construct reflecting the measures operationalised by the previous three latent variables with high correlation coefficients (Table 13).

**Table 13:** Correlations of 2nd order variables.

	Entrepreneurial abilities	Strategic abilities	Technological abilities
Entrepreneurial abilities	1.000		
Strategic abilities	0.794	1.000	
Technological abilities	0.737	0.800	1.000

Instead of five structural relationships, only three will be tested. The first step for assessing our structural model (inner model) is to examine the R^2^ value of the endogenous construct: surviving during the violent covid-19 crisis. The variance explained by the model is about 20%. This measure is critical to assess the model’s explanatory power. Despite its lower score, we consider it acceptable because of the pandemic context of data collection.

**Fig 4:**
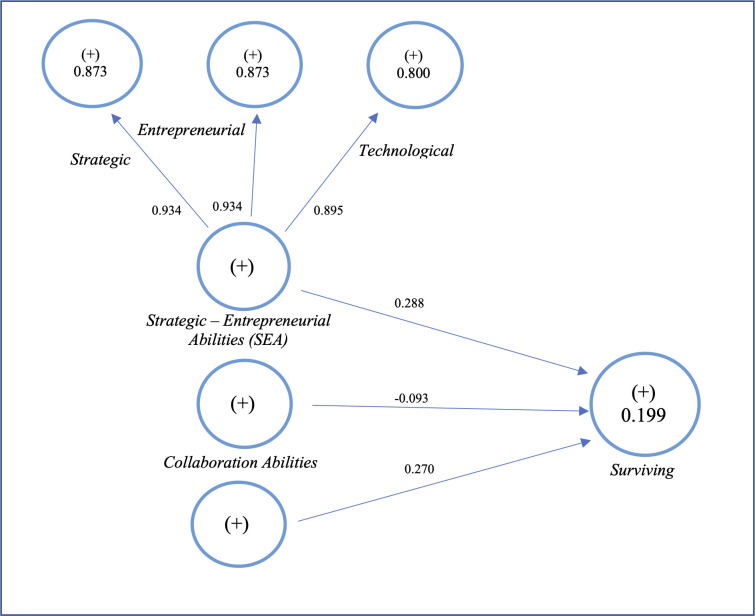
Structural model.

The results provided by blindfolding indicate a lower predictive accuracy (Q^2^ = 0,123; Table 14 for surviving as an endogenous construct). The R^2^ measures the variance explained in each endogenous construct [138]. It explains 20% of the variance of the model (Fig 4). Although it is not substantial because of its value of less than 0.25, we keep it as an actual result in our research context. The interpretation of R^2^ depends on the context. In some topics, an R^2^ value lower than 0.10 is considered acceptable, for example, when predicting with a high level of uncertainty, such as in the Covid-19 context.

**Table 14:** The second-order model’s predictive relevance.

	*SSO*	*SSE*	*Q^2^ (= 1-SSE/SSO)*
*Entrepreneurial ability*	678.000	186.002	0.726
*Relational Abilities*	678.000	678.000	
*Collaboration abilities*	678.000	678.000	
*Strategic Abilities*	678.000	292.556	0.569
*Strategic-Entrepreneurial Abilities*	1808.000	1808.000	
*Surviving*	452.000	396.465	0.123
*Technological Abilities*	452.000	165.288	0.634

Moreover, the R^2^ values depend on the number of explanatory variables (predictors). The more predictor constructs are, the higher the R^2^ value [139]. Our structural model has been limited to three kinds of abilities as predictors (Fig 3).

Based on Fig 4, we can conclude that surviving during the crisis depends more on strategic-entrepreneurial abilities (SEA). The relationship is statistically significant with T_value, given by bootstrapping technique, higher than 1.96. More importantly, the ability to shift resources quickly (Ability 2), to effectively organise the work in the firm and its strategic planning (Ability 3) as well, and its ability to diversify its products and services (Ability 1) are perceived as critical. Similarly, entrepreneurial abilities—which refer to the ability to identify and exploit the opportunities appearing during the crisis (Ability 9) in addition to other abilities such as innovation (Ability 10) and starting a new business (Ability 11)—are seen as strategic assets. Then, they positively impact the firms’ survival when dealing with the violent black swan event. Indeed, technological abilities impact the surviving during a crisis but not to the same degree as entrepreneurial and strategic ones. An efficient IT system (Ability 5) and high technological advancement (Ability 4) are imitable and accessible to everyone. That’s why the latter kind of ability (Technological = 0.895) is perceived as less important than the former (Strategic = Entrepreneurial = 0.934).

The structural model validates another relationship in which relational abilities impact the firm’s survival during the crisis. The coefficient γ = 0,270, and the T_value is more than 1,96, which means the relationship is statistically significant. This result shows that partnering is vital during a crisis, especially with banks. It helps get easy access to financing sources (Ability 12). However, relational abilities might refer to changing suppliers quickly (Ability 13) because the crisis might also impact them. Similarly, firms which can find new customers (Ability 14) have more chances to survive than others.

Regarding relational and entrepreneurial-strategic abilities, collaboration abilities are unlikely to impact survival during the Covid_19 crisis. This result can be explained by the items used to operationalise the construct.

## 5. Conclusions

### 5.1. Problem and methodology summary

This paper sought to understand which organisational abilities influence the company’s survival during crises, specifically during the Covid-19 pandemic turbulence. First, we identified strategically critical organisational skills and capabilities that seem crucial during various crises. Then, using survey and quantitative methods, we analysed which organisational skills and capabilities affected the organisational goals seen by managers as strategically significant that assure further company survival. The research was carried out between November 2020 and February 2021, during the second wave of the Covid-19 pandemic. As a result, we were able to identify the organisational abilities necessary for surviving the corona crisis.

In our study, we have focused on organisations from two countries on two continents that were equally disturbed by the Covid-19 pandemic.

### 5.2. Results summary and discussion

From the carried-out analyses, we can conclude that the company’s survival depends mostly on strategic and entrepreneurial abilities during the crisis. Indeed, strategic thinking during radical and discontinuous changes and determination in decision-making has already been found critical in emergency crises [36, 46, 54, 140]. Our study has specifically demonstrated that, by large, the abilities to shift resources quickly, to organise work effectively, to plan strategically [52], and to diversify the portfolio of goods and services [15] are perceived as the most important strategic manoeuvres.

Other research results indicate that entrepreneurial skills help companies overcome crises [141] and that companies, in order to survive, prepare entrepreneurial responses to crises [94, 95, 102]. Entrepreneurial abilities, seen as the competence to identify and exploit the opportunities appearing during a crisis, are considered strategic assets [39, 40]. What is more, absorptive capacity plays an important role in enhancing the level of entrepreneurship [85]. Similarly, our study shows that using knowledge from the environment [32] for partnering and building social capital play a significant role during the crisis, as it can help get access to financing easier [104], changing suppliers more flexibly [118, 119], and finding new customers [59, 93].

The contribution of this paper is two-fold. First, it adds to the RBV and dynamic capabilities literature by demonstrating which organisational resources and abilities shape survival-oriented strategic choices during a crisis. Second, with relatively little empirical research carried out into crisis responses analysed jointly in various parts of the world (Central and Eastern Europe and North West Africa in our study) during the Covid-19 pandemic, it can be expected that the studies from the two countries can be a starting point for some emerging worldwide patterns. Their economic impact will produce substantially more research in this area.

### 5.3. Implications for theory

The results of our study have several implications for theory. First, they broaden the dynamic capability view by indicating that shifting resources quickly, organising work, planning strategically, diversifying, and building social capital are the capabilities that matter during radical and discontinuous changes. Second, they confirm that the absorptive capacity view is a valid foundation for studying organisations in crises. During unexpected and surprising shocks, it is crucial how the organisations use and benefit from the external knowledge, from the information accessible in the environment, to exploit opportunities, minimise threats and, as a result, learn from the crisis and develop resilience. Third, our analyses clearly highlight the role of both strategic orientation and entrepreneurial orientation of the firm in surviving difficult times. Strategic orientation is often studied in well-functioning organisations as creating the future, less often as a response to the crisis [54]. Entrepreneurial orientation, on the other hand, although proactiveness is one of its dimensions, typically boils down to preparing strategies helping to exploit opportunities ahead of the competition [40]. Again, crafting strategies as responses to crises and exploiting new opportunities due to discontinuous change can be a novel approach to studying the firm’s entrepreneurial orientation.

### 5.4. Implications for practice

The results of our study also signal some managerial implications. First, they highlight the role of regular strategic planning, understood as long-term plans of the enterprise with particular attention to crisis situations, and preparing crisis-readiness plans. Second, by identifying the ability to shift the most important resources quickly, they call for more managerial and strategic flexibility. Such flexibility can be implemented by the following sequence of activities that captures the capabilities we have identified: scanning the environment for opportunities, finding creative ideas, preparing innovations even in crisis, diversifying the product portfolio (i.e. avoiding all eggs in one basket) and creating new business ventures. The final implication focuses on doing it all together: partnering with stakeholders will definitely guide organisations smoother through every crisis. To summarise the managerial implications, it is important for the company to survive during crises to develop the managerial competence to see opportunities rather than threats, to create positive relations with stakeholders, to look for new customers and suppliers and to focus on technological and organisational innovation and creativity for the possibility to offer new services and goods. Finally, in the planning process, access to critical resources needs to be secured from different locations.

### 5.5. Limitations and future research directions

This study has certain limitations which open up avenues for possible future research: method limitations and sample limitations. First, we used a survey with a scale measuring managerial perceptions about capabilities and strategic goals. It would be interesting to measure the actual goals realised by companies before and after the crisis, which would require a longitudinal approach. Second, the list of skills and capabilities is not exhaustive. We purposefully limited it to the bundle of capabilities indicated in the literature regarding crises. The future survey could be designed based on a standardised but exploratory, bottom-up list of capabilities created by managers from scratch without looking at the existing literature. Third, the interest in organisational capabilities led us to two specific geographical and cultural contexts (Poland in Europe and Morocco in Africa). Other geographical or cultural contexts may have resulted in different outcomes. Future research could compare other regions or countries to test whether they are strategic and entrepreneurial.

Future research could be carried out on a larger sample of countries and concentrate on a specific sector or groups of similar sectors to identify resource bundles and capabilities patterns in a crisis.
